# Brain fog in neuropathic postural tachycardia syndrome may be associated with autonomic hyperarousal and improves after water drinking

**DOI:** 10.3389/fnins.2022.968725

**Published:** 2022-08-05

**Authors:** Belén Rodriguez, Annie Hochstrasser, Philippe J. Eugster, Eric Grouzmann, René M. Müri, Werner J. Z’Graggen

**Affiliations:** ^1^Department of Neurosurgery, Inselspital, Bern University Hospital, University of Bern, Bern, Switzerland; ^2^Department of Neurology, Inselspital, Bern University Hospital, University of Bern, Bern, Switzerland; ^3^Service of Clinical Pharmacology, Lausanne University Hospital and University of Lausanne, Lausanne, Switzerland; ^4^Gerontechnology and Rehabilitation Group, ARTORG Center for Biomedical Engineering Research, University of Bern, Bern, Switzerland

**Keywords:** neuropsychology, cognition, stress, autonomic neuropathy, autonomic dysfunction, dysautonomia

## Abstract

**Background:**

Brain fog is a common and highly disturbing symptom for patients with neuropathic postural tachycardia syndrome (POTS). Cognitive deficits have been measured exclusively in the upright body position and mainly comprised impairments of higher cognitive functions. The cause of brain fog is still unclear today. This study aimed to investigate whether increased autonomic activation might be an underlying mechanism for the occurrence of brain fog in neuropathic POTS. We therefore investigated cognitive function in patients with neuropathic POTS and a healthy control group depending on body position and in relation to catecholamine release as a sensitive indicator of acute stress. The second aim was to test the effect of water intake on cardiovascular regulation, orthostatic symptoms, cognitive function and catecholamine release.

**Methods:**

Thirteen patients with neuropathic POTS and 15 healthy control subjects were included. All participants completed a total of four rounds of cognitive testing: two before and two after the intake of 500 ml still water, each first in the supine position and then during head-up tilt. At the end of each cognitive test, a blood sample was collected for determination of plasma catecholamines. After each head-up tilt phase participants were asked to rate their current symptoms on a visual analogue scale.

**Results:**

Working memory performance in the upright body position was impaired in patients, which was associated with self-reported symptom severity. Patients had elevated plasma norepinephrine independent of body position and water intake that increased excessively in the upright body position. The excessive increase of plasma norepinephrine was related to heart rate and symptom severity. Water intake in patients decreased norepinephrine concentrations and heart rate, and improved symptoms as well as cognitive performance.

**Conclusion:**

Brain fog and symptom severity in neuropathic POTS are paralleled by an excessive norepinephrine secretion. Bolus water drinking down-regulates norepinephrine secretion and improves general symptom severity including brain fog.

## Introduction

Postural tachycardia syndrome (POTS) is a multi-system disorder of the autonomic nervous system, and is characterized by chronic symptoms of orthostatic intolerance. Besides the presence of orthostatic symptoms for at least 3 months, diagnostic criteria require a clinically symptomatic sustained heart rate increment of 30 beats or more per minute or an increase in heart rate to ≥ 120 beats per minute within 10 min of standing or head-up tilt while maintaining blood pressure control (Vernino et al., 2021). Different etiologies of POTS have been described. Neuropathic POTS is the most common type and is thought to be caused by peripheral sympathetic noradrenergic denervation leading to increased venous pooling (Jacob et al., 2000; Peltier et al., 2010). In recent years, an increasing number of studies reported the presence of autoantibodies against G-coupled protein receptors in patients with neuropathic POTS, suggesting an immune-mediated pathogenesis in a subset of these patients (Li et al., 2014; Fedorowski et al., 2017; Ruzieh et al., 2017; Watari et al., 2018; Gunning, et al., 2019). Other proposed pathophysiological mechanisms include abnormally increased sympathetic activity and circulating catecholamine excess (hyperadrenergic type; Schondorf and Low, 1993; Jacob and Biaggioni, 1999), and absolute hypovolemia due to low blood volume (Vernino et al., 2021).

A common and strongly disturbing symptom for patients is difficulty concentrating (Deb et al., 2015; Shaw et al., 2019). Patients often have problems to accurately describe their cognitive difficulties, and usually refer to them as “brain fog” or “mental clouding” (Ross et al., 2013; Wells et al., 2020). Ross et al. conducted an online questionnaire-based study investigating the symptom of brain fog, and found that the most commonly used descriptors of brain fog were “forgetful,” “cloudy,” and “difficulty focusing, thinking and communicating” (Ross et al., 2013). Many studies have shown that cognitive dysfunction is measurable in patients with POTS, and is most evident in higher cognitive functions such as executive function, particularly working memory performance (Ocon et al., 2012; Stewart et al., 2012, 2015; Anderson et al., 2014), but also in selective and sustained attention (Raj et al., 2009; Anderson et al., 2014; Arnold et al., 2015), and cognitive processing speed (Ocon et al., 2012; Arnold et al., 2015). Importantly, all measured cognitive deficits were limited to the upright posture. While the literature is fairly consistent regarding the type of cognitive dysfunction in POTS, the cause of this symptom remains unclear and controversial. Initially, orthostatic cerebral hypoperfusion was assumed to be responsible for the occurrence of cognitive symptoms. This is highly unlikely since cerebral perfusion primarily depends on (i) mean arterial blood pressure, (ii) intracranial pressure and (iii) cerebral autoregulation (Le Roux et al., 2014; Hani et al., 2021), and all these factors are unaffected in POTS: first, the diagnostic criteria for POTS presuppose normal blood pressure regulation; second, the regulation of intracranial pressure in POTS is not affected by orthostasis (Cipriani et al., 2019); and third, patients with POTS have intact cerebral autoregulation (Schondorf et al., 2005; Endo et al., 2014; Wells et al., 2020). Other studies suggested that the impairment in cognitive performance in POTS may occur when attention is directed to somatic sensations due to increased internal vigilance when patients are symptomatic (Anderson et al., 2014; Owens et al., 2018a,b). Finally, central norepinephrine dysregulation is thought to be associated with cognitive dysfunction, particularly in relation to the hyperadrenergic subtype of POTS (Arnold et al., 2015; Raj et al., 2018).

In our previous study, we confirmed that working memory performance is impaired in neuropathic POTS during head-up tilt compared to the supine position, as well as compared to the performance of healthy subjects, and showed that this deficit improved after ingestion of half a liter of water (Rodriguez et al., 2019). The improvement in cognitive performance was paralleled by a reduction in heart rate increase and subjective symptom perception, both known effects of water intake in POTS (Jacob et al., 1997; Gordon et al., 2000; Shannon et al., 2002; Z’Graggen et al., 2010; Snapper and Cheshire, 2022). Fittingly, Ross and colleagues showed that 86% of patients reported reduced fluid intake as a trigger of cognitive dysfunction and that treatment with intravenous saline improved cognitive performance (Ross et al., 2013). Water intake in neuropathic POTS appears to improve peripheral sympathetic functioning, leading to a reduction in peripheral blood pooling, heart rate increase and ultimately in subjectively perceived orthostatic stress (Scott et al., 2001; Jordan, 2005; Lu et al., 2012). A growing body of research indicates that acute stress (physical as well as psychosocial) in healthy individuals leads to impairments in working memory and cognitive flexibility *via* sympathetic nervous system activation (Elzinga and Roelofs, 2005; Buchanan et al., 2006; Alexander et al., 2007; Schoofs et al., 2008, 2009; Marko and Riecansky, 2018). Based on these findings and the results of our previous study, we hypothesized that increased sympathetic stress and consequently increased autonomic arousal (“hyperarousal”) is an important underlying mechanism for the occurrence of orthostatic cognitive dysfunction in neuropathic POTS. To address this hypothesis, we conducted the present study and investigated orthostatic cognitive deficits in neuropathic POTS in relation to catecholamine release as a sensitive indicator of acute stress (Glavin, 1985; Dimsdale et al., 1987; Morilak et al., 2005; Bali and Jaggi, 2015). The second aim of this study was to confirm our previous findings regarding water intake in patients with neuropathic POTS in a larger sample and additionally to test the effect of water intake on catecholamine release.

## Materials and methods

### Participants

All procedures of the present study received local ethical approval (Kantonale Ethikkommission Bern, Switzerland) and were carried out in accordance with the Declaration of Helsinki and its amendments. All study procedures were performed at the University Hospital Bern, Inselspital, Switzerland. Fifteen patients with confirmed neuropathic POTS and 15 healthy control subjects participated in this study. Written informed consent was given by all participants. Exclusion criteria for all participants were pregnancy, breast feeding and age ≤ 18 or ≥ 60 years. An additional exclusion criterion for patients was if their current medication could not be discontinued due to clinical reasons. Further exclusion criteria for control subjects included arterial hypertension, vasovagal syncope in medical history and intake of vasoactive medications. Diagnosis of neuropathic POTS was based upon medical history, physical and neurological examination, cardiovascular autonomic function testing, thermoregulatory sweat test and/or quantitative testing of sudomotor axon reflexes, measurement of plasma norepinephrine concentrations, determination of autoantibodies against G-protein-coupled receptors, and in some cases skin biopsies. Participants had no behavioral or dietary restrictions in the days prior to the examination, but were not allowed to eat or drink after midnight on the day of the examination. In patients, all vasoactive and psychoactive medication was stopped at five half-lives prior to testing.

### Material

#### Questionnaires

All participants were asked to complete the following self-report questionnaires in German the day before the examination: Beck Depression Inventory Second Edition (BDI-II; Beck and Steer, 1984), Pittsburgh Sleep Quality Index (PSQI; Buysse et al., 1989) and 36-Item Short Form Health Survey (SF-36; Ware and Sherbourne, 1992).

#### Cardiovascular autonomic function testing

All participants were placed comfortably in the supine position on a tilt table. Throughout the experimental procedure beat-to-beat blood pressure and heart rate were continuously recorded with the Finapres® NOVA device (Finapres Medical Systems BV, Arnhem, the Netherlands) from the non-dominant hand. In addition, a three-lead electrocardiogram was recorded and intermittent brachial blood pressure and heart rate values were measured using a Mindray VS-900 sphygmomanometer (ITRIS Medical AG, Spreitenbach, Switzerland) on the non-dominant arm. Testing was started after heart rate and blood pressure remained stable for 15 min in the supine position. Head-up tilt testing was performed with a tilt angle of 60° for 15 min.

#### Cognitive testing

For cognitive testing the subtest “Sustained Attention” of the computer-based test battery “Test of Attentional Performance” (TAP, version 2.3.1) was used. The duration of this test is 15 min. The subtest “Sustained Attention” was selected because it requires maintenance of attention on a mentally demanding activity for a sustained period of time, which is a typical requirement in working life. This subtest primarily tests the function of working memory, but also requires sustained and selective attention. During the test a sequence of stimuli is presented on a monitor. The stimuli vary in four feature dimensions: color, shape, size and filling. A target stimulus occurs whenever it matches the preceding stimulus in either color or shape. Overall, 450 stimuli were presented at two second intervals, including 54 targets. The test was implemented in the highest level of difficulty (“color or shape”). The parameters median reaction time (milliseconds), and number of omissions (=no response to a target stimulus) were recorded, and given as absolute values and percentile ranks. The participants responded by pressing a button placed in their dominant hand. A total of four rounds of cognitive testing per participant were completed (please see section “Study protocol”).

#### Assessment of plasma norepinephrine and epinephrine concentrations

After installation of the devices for cardiovascular autonomic function testing, an intravenous 18G-catheter was inserted into the participant’s antecubital vein of their dominant arm to repeatedly measure plasma norepinephrine and epinephrine concentrations. To prevent clogging of the intravenous catheter, a 0.9% saline infusion was administered at a rate of 5 ml/h *via* a syringe pump. During blood collection, the syringe pump was paused. A first blood sample of 7.5 ml was taken and discarded to avoid dilution by the saline infusion administered. The second sample of 4.7 ml was used for the analysis. A total of four blood samples were taken per participant to determine plasma concentrations of norepinephrine and epinephrine (please see section “Study protocol”). The blood samples were immediately centrifuged at 4°C at 2,500 RCF and the plasma frozen at the Center for Laboratory Medicine of the University Hospital Bern, Inselspital, Switzerland. The analysis was performed at the Laboratory for Catecholamines of the Lausanne University Hospital CHUV, Switzerland. The concentrations of norepinephrine and epinephrine in plasma were measured by ultra-high pressure liquid chromatography tandem mass spectrometry (UHPLC-MS/MS). The method was the same as described in (van Faassen et al., 2020), except that the online SPE was replaced by an off-line SPE using Oasis HLB 5 mg 96-well plates. See Supplementary material for details on the method and the validation.

### Study protocol

**FIGURE 1:**
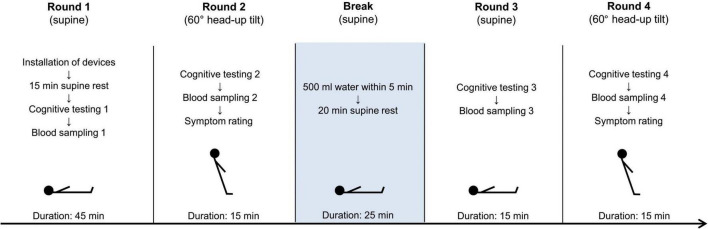
Study protocol. Participants completed four rounds of cognitive testing (test “Sustained Attention” from the test battery “Test of Attentional Performance”). Blood samples for catecholamine assessment were taken four times, each time in the last minute of the test. After each head-up tilt phase, all participants rated ten of the most frequent symptoms of orthostatic intolerance.

The detailed study protocol is shown in Figure 1. The examination took place in a standardized and controlled environment in terms of light, temperature and humidity. Cognitive testing was started after heart rate and blood pressure remained stable for 15 min in the supine position. To ensure a calm environment the lights were dimmed and interactions with the participants were kept to a minimum. Participants were familiarized with the cognitive test before completing the first round of testing in the supine position. After a five-minute rest in the supine position, the same test was then repeated in the upright (60° head-up tilt) position. After a short recovery in the supine position, participants were asked to drink 500 ml of commercially available still mineral water (Evian ®, Société anonyme des eaux minérales d’Évian, Évian-les-Bains, France) at room temperature within 5 min. After 20 min of supine rest, cognitive testing was repeated as described above in the supine and upright position. The intervention of rapid water drinking was performed according to an established protocol (Scott et al., 2001; Jacob et al., 2019; Rodriguez et al., 2019). Blood samples were always taken 14 min after the start of the cognitive test without previous notice. Participants were asked to rate their subjective perception of the most frequent symptoms of orthostatic intolerance (difficulty concentrating, dizziness, lightheadedness, orthostatic headache, nausea, weakness, palpitations, blurred vision, shortness of breath, and fatigue) on a 10-point visual analogue scale after each head-up tilt phase (symptom rating; maximum sum score 100).

### Data analysis

Statistical analyses were performed using Statistical Package for the Social Sciences (SPSS Statistics) Version 25.0 (IBM Corp., Armonk, NY, United States). In case of normally distributed data parametric testing and for non-normally distributed variables non-parametric testing was implemented. Group differences in patient characteristics and questionnaires were assessed using two tailed Student’s *t*-test for independent samples, Mann–Whitney *U*-test and Fisher’s exact test, respectively. Changes in symptom ratings (for sum of all symptom ratings and difficulty concentrating separately) were analyzed using two-way analyses of variance (ANOVA) for repeated measures with *post hoc* Bonferroni correction for multiple comparisons. The factors were i) group with two levels (patients; control subjects) and ii) water intake with two levels (before; after). For the analysis of heart rate, blood pressure, cognitive performance as well as plasma norepinephrine and epinephrine concentrations, three-way ANOVA for repeated measures with *post hoc* Bonferroni correction for multiple comparisons were conducted. The factors were i) group with two levels (patients; control subjects), ii) body position with two levels (supine; head-up tilt) and iii) water intake with two levels (before; after). The analysis of heart rate and blood pressure was based on mean values of the continuously measured values with the Finapres NOVA device during the last minute before the start of the head-up tilt and during the last minute of the head-up tilt, respectively. The analysis of reaction time was based on individual median values. Omissions were processed as absolute values. Pearson’s correlations were exploratively applied to investigate possible associations between the following parameters: (changes in) norepinephrine concentrations, (changes in) epinephrine concentrations, (changes in) heart rate, (changes in) subjective symptom perception and (changes in) cognitive performance (omissions and reaction time). Group data are reported as mean (±standard deviation). A two-tailed *p* value ≤ 0.05 was defined as statistically significant.

## Results

### Participant characteristics and cardiovascular autonomic function

Tables 1 and 2 summarize the participant’s demographic data including the results of the questionnaires, the heart rates and blood pressures in supine and head-up tilt positions before and after water intake as well as the symptom ratings. Two patients were excluded from all analyses due to the occurrence of pre-syncope during the first head-up tilt phase, resulting in a final sample size of 15 healthy subjects and 13 patients with POTS. In one patient and one healthy subject blood sampling was not possible due to a clogged catheter. Thus, the sample size for analyses of plasma norepinephrine and epinephrine was 14 healthy subjects and 12 patients with POTS. The groups were matched for age and sex. In ten out of 13 patients (77%) autoantibodies against G-protein coupled receptors are present, and in two patients (15%) the skin biopsy is pathological. One patient (0.8%) has additional celiac disease, nine patients (70%; eight of whom have autoantibodies) have gastrointestinal dysmotility, and two patients (15%) are on the hypermobility spectrum without meeting the diagnostic criteria for Ehlers-Danlos syndrome. Patients with POTS scored significantly higher than healthy subjects in the Beck Depression Inventory, the Pittsburgh Sleep Quality Index, and in the physical health summary scale of the Health Survey SF-36. The mental health summary scale of the Health Survey SF-36 was not significantly different between the two groups. The analyses for symptom ratings showed a main effect for water intake (for symptom ratings overall: *F*(1, 26) = 13.79, *p* = 0.001) and significant interactions of water intake*group (for symptom ratings overall: *F*(1, 26) = 6.42, *p* = 0.018; for difficulty concentrating: *F*(1, 26) = 5.95, *p* = 0.022). Pairwise comparisons revealed that during both head-up tilt phases, patients experienced more symptoms of orthostatic intolerance in general, and specifically also more difficulty concentrating than healthy subjects (all *p* < 0.001). After the intake of water, patients had significantly less symptoms overall, and specifically also less difficulty concentrating during head-up tilt than before water intake (*p* < 0.001 for symptoms overall; *p* = 0.008 for difficulty concentrating). No such effect was observed for healthy subjects (*p* = 0.331 for symptoms overall; *p* = 0.639 for difficulty concentrating). The analysis of heart rate revealed main effects for body position [*F*(1, 26) = 218.37, *p* < 0.001] and water intake [*F*(1, 26) = 9.24, *p* = 0.005] as well as a significant interaction of body position*group [F(1, 26) = 22.83, *p* < 0.001]. Pairwise comparisons showed that the heart rate increased from the supine to the head-up tilt position in both groups before as well as after water intake (all *p* < 0.001). During both head-up tilt phases, patients had a higher heart rate than healthy subjects (both *p* < 0.001), but the heart rate of patients was significantly lower after water intake (*p* = 0.022). No such difference was found in the control group (*p* = 0.362). The analyses for systolic and diastolic blood pressure did not reveal any significant main effects or interactions. Systolic and diastolic blood pressure did not differ between the groups at any time.

**TABLE 1:** Participant characteristics and symptoms during head-up tilt.

	Control (*N* = 15)	POTS (*N* = 13)	*p* value*
Age	28.13 (±2.93)	27.62 (±5.95)	0.786
Sex (female)	13 (87%)	11 (85%)	0.644
BDI-II	3.67 (±3.66)	11.92 (±8.42)	0.001
PSQI	3.47 (±1.89)	8.15 (±4.16)	0.001
SF-36 physical health	56.88 (±4.49)	36.17 (±6.83)	<0.001
SF-36 mental health	49.53 (±7.53)	47.59 (±9.43)	0.786
Symptom rating before water	7.80 (±6.97)	50.46 (±10.27)	<0.001
Symptom rating after water	5.53 (±6.51)	39.69 (±14.35)	<0.001
Difficulty concentrating before water	0.6 (±0.99)	6.85 (±1.66)	<0.001
Difficulty concentrating after water	0.8 (±1.24)	5.54 (±1.81)	<0.001

Data are reported as mean (± standard deviation) if not differently indicated.

*p values are referring to group differences.

POTS, postural tachycardia syndrome; BDI-II, Beck Depression Inventory Second Edition; PSQI, Pittsburgh Sleep Quality Index; SF-36, 36-Item Short Form Health Survey.

**TABLE 2:** Results of cardiovascular autonomic function testing.

		Supine before water intake	Tilt before water intake	Supine after water intake	Tilt after water intake
Heart rate (bpm)	Control (*N* = 15)	63.73 (±8.07)	80.13 (±11.78)	59.20 (±5.63)	78.20 (±9.32)
	POTS (*N* = 13)	66.63 (±13.55)	103.23 (±15.74)	65.15 (±10.56)	97.77 (±16.05)
Systolic blood pressure (mmHg)	Control (*N* = 15)	118 (±12.31)	114.6 (±11.67)	116.4 (±12.55)	120.67 (±11.64)
	POTS (*N* = 13)	114.85 (±9.35)	111.92 (±14.83)	116.15 (±9.16)	113.54 (±16.47)
Diastolic blood pressure (mmHg)	Control (*N* = 15)	77.53 (±9.17)	82.2 (±7.93)	73.67 (±10.78)	81.87 (±9.76)
	POTS (*N* = 13)	72.69 (±6.29)	77.46 (±13.69)	74.31 (±7.04)	76.38 (±10.18)

Data are reported as mean (± standard deviation). Heart rate and blood pressure were measured in the supine position just before head-up tilt and 15 min after the start of head-up tilt. POTS, postural tachycardia syndrome; bpm, beats per minute.

### Cognitive testing

**FIGURE 2:**
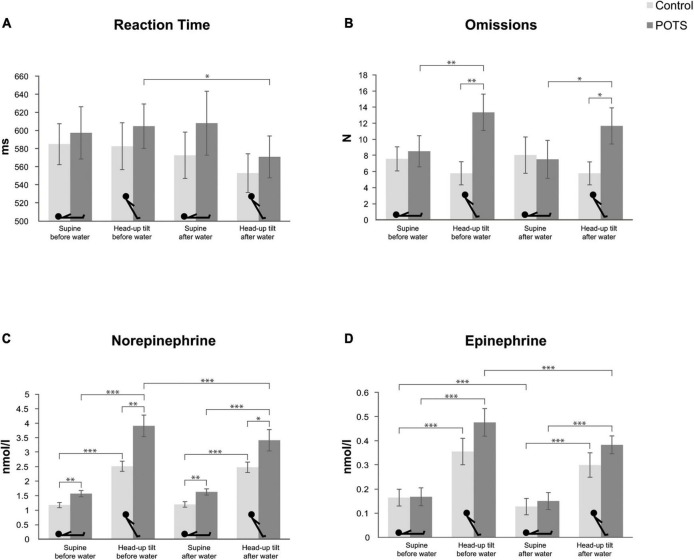
Results of cognitive testing and assessment of plasma norepinephrine and epinephrine. **(A)** Bar graphs showing the reaction times of patients with neuropathic POTS (dark gray bars) and control subjects (light gray bars) in the supine and 60° head-up tilt position, each before and after the intake of 500 ml water. The same is shown for **(B)** omissions, **(C)** plasma norepinephrine concentrations, and **(D)** plasma epinephrine concentrations. Values are given as means ± S.E.M. *p ≤ 0.05, ^**^p ≤ 0.01, ^***^p ≤ 0.001.

The results of cognitive testing including percentile ranks for each parameter are reported in Table 3 and illustrated in Figures 2A,B. For reaction time, there was a significant interaction of body position*water intake [*F*(1, 26) = 4.82, *p* = 0.037]. Pairwise comparisons showed that reaction time during head-up tilt was significantly faster in patients with POTS after the intake of water than before water intake (*p* = 0.042). For omissions, the analysis showed a significant interaction of body position*group [*F*(1, 26) = 19.46, *p* < 0.001]. Pairwise comparisons revealed that during both head-up tilt phases patients made more omissions than healthy subjects (before water: *p* = 0.007; after water: *p* = 0.032), and that patients made more omissions during head-up tilt than in the supine position, both before (*p* = 0.001) and after water intake (*p* = 0.011). No such changes were found for healthy subjects.

**TABLE 3:** Results of cognitive testing and assessments of plasma catecholamines.

		Supine before water intake	Tilt before water intake	Supine after water intake	Tilt after water intake
Reaction time (ms)	Control (*N* = 15)	585.13 (±84.61)	582.80 (±96.86)	572.80 (±95.94)	553.13 (±79.99)
	POTS (*N* = 13)	597.62 (±100.01)	604.92 (±85.17)	608.31 (±122.07)	571.08 (±79.97)
Percentile rank reaction time	Control (*N* = 15)	49.20 (±24.38)	50.93 (±25.95)	53.80 (±26.18)	59.13 (±22.40)
	POTS (*N* = 13)	46.46 (±28.36)	43.92 (±21.89)	45.77 (±30.49)	51.62 (±24.92)
Omissions (*N*)	Control (*N* = 15)	7.60 (±5.60)	5.80 (±5.38)	8.07 (±8.46)	5.80 (±5.36)
	POTS (*N* = 13)	8.54 (±6.71)	13.39 (±7.84)	7.54 (±8.16)	11.69 (±7.78)
Percentile rank omissions	Control (*N* = 15)	51.20 (±25.21)	61.80 (±28.85)	58.86 (±31.60)	60.60 (±28.26)
	POTS (*N* = 13)	49.69 (±31.41)	32.83 (±32.24)	58.00 (±35.90)	36.15 (±28.99)
Norepinephrine (nmol/l)	Control (*N* = 14)	1.18 (±0.32)	2.52 (±0.63)	1.20 (±0.34)	2.48 (±0.65)
	POTS (*N* = 12)	1.57 (±0.35)	3.91 (±1.24)	1.63 (±0.36)	3.42 (±1.23)
Epinephrine (nmol/l)	Control (*n* = 14)	0.17 (±0.13)	0.36 (±0.20)	0.13 (±0.12)	0.30 (±0.18)
	POTS (*n* = 12)	0.17 (±0.12)	0.48 (±0.19)	0.15 (±0.12)	0.38 (±0.12)

Data are reported as mean (± standard deviation). Interpretation of percentile ranks: <3 = clearly below average; 4–15 = below average; 16–34 = lower average; 35–65 = middle average; 66–84 = upper average; 85–97 = above average; >97 = clearly above average. Blood samples for assessment of plasma norepinephrine and epinephrine were always taken during the last minute of cognitive testing (14 min after the start of the test). POTS, postural tachycardia syndrome.

### Plasma norepinephrine and epinephrine concentrations

The results of assessment of plasma norepinephrine and epinephrine concentrations are summarized in Table 3 and shown in Figures 2C,D. The analysis of norepinephrine showed main effects of body position [*F*(1, 24) = 89.70, *p* < 0.001], water intake [*F*(1, 24) = 6.63, *p* = 0.017], and group [*F*(1, 24) = 11.95, *p* = 0.002]. Furthermore, there were significant two-way interactions of body position*group [*F*(1, 24) = 4.46, *p* = 0.045], body position*water intake [*F*(1, 24) = 16.38, *p* < 0.001], and water intake*group [*F*(1, 24) = 5.79, *p* = 0.024], as well as a three-way interaction of body position*water intake*group [*F*(1, 24) = 10.78, *p* = 0.003]. Pairwise comparisons revealed that norepinephrine concentrations were higher in each measurement in patients compared to the control group (supine before water: *p* = 0.008; head-up tilt before water: *p* = 0.006; supine after water: *p* = 0.002; head-up tilt after water: *p* = 0.026). Furthermore, norepinephrine concentrations increased during head-up tilt in patients with POTS and healthy subjects both before and after water intake (all *p* < 0.001). However, this increase in norepinephrine concentrations before water intake was more pronounced in patients than in healthy subjects (increase of 2.34 nmol/l vs. 1.34 nmol/l; *p* = 0.012). Only patients had significantly lower norepinephrine concentrations during head-up tilt after compared to before water intake (*p* < 0.001). Norepinephrine concentrations during head-up tilt exceeding 3.55 nmol/l (equivalent to 600 pg/ml) were present in 67% (8/12) of the patients and in none of the control group. The analysis for epinephrine showed main effects for body position [*F*(1, 24) = 64.05, *p* < 0.001] and water intake [*F*(1, 24) = 11.23, *p* = 0.002]. Pairwise comparisons showed that, same as for norepinephrine concentrations, epinephrine concentrations increased during head-up tilt for both groups in both conditions (all *p* < 0.001). Additionally, in healthy subjects, epinephrine concentrations were decreased in the supine position after water intake (*p* = 0.006), and in patients with POTS in the head-up tilt position after water intake (*p* = 0.041).

### Exploratory correlation analyses

The exploratory correlation analyses showed that during head-up tilt symptom ratings positively correlated with heart rate (before water intake: *r* = 0.697, *R*^2^ = 0.486, *p* < 0.001; after water intake: *r* = 0.666, *R*^2^ = 0.443, *p* < 0.001) and omissions (before water intake: *r* = 0.484, *R*^2^ = 0.234, *p* = 0.009; after water intake: *r* = 0.515, *R*^2^ = 0.266, *p* = 0.005). Norepinephrine concentrations during head-up tilt were associated with heart rate (before water intake: *r* = 0.488, *R*^2^ = 0.238, *p* = 0.011) and symptom ratings (before water intake: *r* = 0.603, *R*^2^ = 0.364, *p* = 0.001; after water intake: *r* = 0.515, *R*^2^ = 0.051, *p* = 0.005). Furthermore, the decrease in norepinephrine concentrations during head-up tilt after compared to before water intake correlated with the simultaneous decrease in symptom ratings (*r* = 0.499, *R*^2^ = 0.249, *p* = 0.009). There were no significant correlations between (changes in) norepinephrine concentrations and (changes in) cognitive performance. None of the correlation analyses performed with epinephrine were significant: Epinephrine concentrations during head-up tilt were not associated with heart rate (before water intake: *r* = 0.131, *R*^2^ = 0.017, *p* = 0.524) or symptom ratings (before water intake: *r* = 0.351, *R*^2^ = 0.123, *p* = 0.079; after water intake: *r* = 0.285, *R*^2^ = 0.081, *p* = 0.158). The decrease in epinephrine concentrations during head-up tilt after compared to before water intake was not correlated with the decrease in symptom ratings (*r* = -0.172, *R*^2^ = 0.030, *p* = 0.401).

## Discussion

This study investigated the possible association between cognitive dysfunction in POTS and catecholamine release as a sensitive indicator of acute stress, and additionally tested the effect of water intake on these parameters. In summary, the results provide further evidence for the occurrence of pure orthostatic cognitive deficits in POTS and show that patients with POTS generally had higher norepinephrine concentrations with a more pronounced increase during head-up tilt than healthy subjects. After water intake, heart rate, subjective perception of symptoms and norepinephrine concentrations during head-up tilt were significantly lower in patients compared to before water intake, and there was also a tendency for improved cognitive performance.

Although our cohort of patients with POTS had a higher BDI-II score compared to controls, patients did not reach a mean score of 18, which is considered the cut-off value for the presence of clinical depression (Beck and Steer, 1984). Based on this, we conclude that the results of cognitive testing in this study were most probably not affected by cognitive symptoms of depression (Hammar and Ardal, 2009). The mean PSQI score of 8 in patients indicates poor sleep quality (Buysse et al., 1989), which is in line with previous reports on sleep disturbances in POTS (Bagai et al., 2011; Mallien et al., 2014; Miglis et al., 2016). We consider a negative influence of sleep quality on cognition unlikely in this study, as the cognitive performance of patients was normal during both tests in the supine position (before and after water intake). Compared to the control group, patients scored higher in the physical but not the mental part of the health summary scale of the Health Survey SF-36, which reflects the burden of disease for patients with POTS (Anderson et al., 2014; Moon et al., 2016).

During both head-up tilt phases patients showed a clinically symptomatic heart rate increase of more than 30 beats per minute. The measured increase in heart rate during head-up tilt was probably more pronounced due to the ongoing cognitive testing. Nevertheless, the intake of 500 ml water was not only associated with a decrease in heart rate during head-up tilt, but also with a decrease in overall symptoms and specifically also in brain fog (“difficulty concentrating”). Both results confirm previous findings of the beneficial effect of water drinking on orthostatic tachycardia and symptoms of orthostatic intolerance (Jacob et al., 1997; Gordon et al., 2000; Z’Graggen et al., 2010; Rodriguez et al., 2019; Snapper and Cheshire, 2022).

Cognitive testing confirmed that patients with POTS experience cognitive impairment in the upright body position. This is evidenced by the increase in omissions made by patients during head-up tilt compared to when they were supine, indicating a decrease in working memory performance. Consistent with previous findings, only cognitive performance in the head-up tilt position was impaired compared to a healthy control group, thus confirming that cognitive dysfunction in POTS is a functional, and not an absolute deficit (Ocon et al., 2012; Stewart et al., 2015; Rodriguez et al., 2019; Wells et al., 2020). The exploratory correlation analyses showed that cognitive performance during head-up tilt correlated with symptom ratings. This finding suggests that the more symptomatic patients feel, the worse their performance is, and thus also supports the hypothesis that increased internal vigilance when patients are symptomatic may lead to worse cognitive performance due to diverted attention to somatic sensations (Low et al., 2009; Anderson et al., 2014; Owens et al., 2018b). Furthermore, the mean percentile ranks of omissions show that working memory performance of patients with POTS was in the middle average range in the supine positions both before and after water intake. During the first head-up tilt phase, working memory performance was worse and only in the lower average range, whereas during head-up tilt after water intake, working memory performance improved to the middle average range. The reaction times did not differ between healthy subjects and patients, and did not show any position-dependent changes within the groups. In patients, however, the median reaction time during head-up tilt was faster after water intake compared to before water intake, indicating an improved alertness after water ingestion. The mean percentile ranks of reaction time were all in the middle average range.

Overall, water intake had a positive impact on orthostatic cognitive performance, as evidenced by faster reaction times, a less pronounced increase in omissions and better percentile ranks during head-up tilt after water intake compared to before. The positive effect of water intake may also be underestimated in the present study: First, the effect of water intake on cognitive functions might have been negatively influenced by a possible fatigue effect due to the long duration of the experiment. In addition, the cognitive test chosen may have been not challenging enough and therefore not sensitive enough to detect small improvements. The results of previous studies show that orthostatic cognitive deficits in POTS become more apparent with increasing test difficulty (Ocon et al., 2012; Rodriguez et al., 2019).

Plasma norepinephrine concentrations increased from the supine to the upright position in both groups, but in patients this increase was more pronounced, which was also paralleled by a greater heart rate increase and more symptoms. In healthy subjects, norepinephrine concentrations increased by a mean of 1.34 nmol/l, which is in line with reported normal values (Meredith et al., 1992; Thieben et al., 2007), and in patients by a mean of 2.34 nmol/l, which is a greater increase than it has been previously described in this patient group (Thieben et al., 2007). In 67% of patients the norepinephrine concentrations during head-up tilt exceeded 3.55 nmol/l (equivalent to 600 pg/ml), which is an accepted diagnostic criterion for the presence of hyperadrenergic POTS (Schondorf and Low, 1993; Jacob and Biaggioni, 1999; Jacob et al., 2019). Since norepinephrine concentrations were measured during cognitive testing and after a tilt duration of 14 min, we believe that this criterion cannot be applied for the present study. Nevertheless, these findings indicate that patients with neuropathic POTS appear to experience some degree of hyperadrenergic state in the upright position under conditions similar to those in our study, as has been suggested by other research groups (Mar and Raj, 2020). Two factors have to be taken into account for the interpretation of the observed excessive norepinephrine increase in the upright position: (i) compensation for the insufficient vasoconstriction in the lower extremities due to the peripheral sympathetic dysfunction, and (ii) the additional stress due to cognitive testing. The influence of the latter is probably small in the present study, as plasma epinephrine concentrations, reflecting adrenomedullary stimulation induced by mental stress (Wortsman, 2002; Goldstein and Kopin, 2008; Carter and Goldstein, 2015), were not significantly different between the two groups at any time point, although there was a tendency for higher epinephrine concentrations during head-up tilt in patients with POTS. We therefore conclude that in patients with neuropathic POTS, the excessive increase in plasma norepinephrine in the upright position occurs mainly in response to the reduced vasoconstrictor function of the sympathetic nervous system.

Interestingly, while within the reported range of normal values (Pluto and Burger, 1988; Zoccali et al., 2002), absolute norepinephrine concentrations were elevated in patients compared to healthy subjects independent of body position and water intake. This would suggest that patients with POTS have an increased activity of the sympathetic nervous system even when they are lying down, indicating that either some extent of hyperadrenergic state might also be present in the supine position, or that it may take more time than in healthy individuals for the autonomic nervous system to fully regulate after orthostatic stress. Especially with regard to the high frequency of sleep disturbances in POTS, the finding of increased norepinephrine secretion even when patients are lying down seems interesting (Bagai et al., 2011; Mallien et al., 2014; Miglis et al., 2016). In the present study, 77% of patients with POTS had autoantibodies against G-protein coupled receptors. This high number of patients with impaired synaptic transmission within the autonomic nervous system may explain the increased norepinephrine concentrations found also in the supine position as well as the extensive norepinephrine increase in the upright body position. This finding has to be interpreted with caution, as participants in our study were in the supine position for only 45 min before the first blood sample was collected and blood sampling occurred during cognitive testing. However, similar to our results, another research group found that patients with chronic orthostatic intolerance had an overall increased supine noradrenergic tone and a decreased postganglionic sympathetic response to head-up tilt with compensatory cardiac sympathetic over-activity (Furlan et al., 1998). The authors concluded that in this patient cohort, the functional distribution of central sympathetic tone to the heart and vessels is abnormal, and suggested that in addition to peripheral sympathetic dysfunction, alterations in the central nervous system may lead to a hyperadrenergic state. Interestingly, a recent systematic review pointed out that patients with sympathetic over-activity, decreased cardiac vagus drive, and systemic inflammation may benefit from transdermal auricular vagus stimulation (Diedrich et al., 2021). In the context of the results of the present study and the study cited above (Furlan et al., 1998), it is therefore possible that a treatment with transdermal auricular vagus stimulation may also be beneficial for patients with neuropathic POTS.

Additionally, and in patients only, norepinephrine concentrations during head-up tilt significantly decreased after water intake, which was associated with an improvement of symptoms. This finding supports the occurrence of the water-induced pressor response and also its assumed mechanisms (Scott et al., 2001; Jordan, 2005; Lu et al., 2012). In summary, the tone of the sympathetic nervous system appears to be down-regulated after water ingestion, and patients with neuropathic POTS therefore experience less arousal and fewer symptoms during head-up tilt, which in turn could be a reason for better cognitive performance.

The present study must be considered with some limitations. First, the study includes a limited sample size, so that the analysis of complex regression models such as a mediation analysis was not possible. Second, all participants had the same procedure during the examination; the order of the conditions (e.g., supine vs. head-up tilt, before vs. after water) was not randomized. Therefore, although we included a control group, we cannot completely exclude the presence of confounding learning and/or fatigue effects. We chose the present study protocol to avoid the possible confounding effect of the known day-to-day fluctuation of symptoms in patients with POTS. Additionally, only patients with neuropathic POTS were included in the study, leading to limited generalizability of the results with respect to other POTS subtypes, especially hyperadrenergic POTS. Previous studies have shown that muscle sympathetic nerve activity peaks 30 min after water intake and that plasma norepinephrine concentrations change at around the same time (Jordan et al., 1999; Scott et al., 2001). The second half of the experiment was therefore started 20 min after water intake to ensure that the expected peak of the water effect occurred during the phase of cognitive testing and catecholamine assessment. Furthermore, the heterogeneity of the underlying etiologies and comorbidities of POTS in our cohort, particularly the presence of gastrointestinal dysmotility, may have had an impact on the results regarding water intake (Dipaola et al., 2020). Finally, the described excessive increase in plasma norepinephrine concentration may not necessarily represent an increase in central sympathetic activity but also a decrease in norepinephrine clearance, as plasma norepinephrine concentration is the result of the balance between norepinephrine release and clearance. The inclusion of indices of cardiovascular autonomic control such as heart rate and blood pressure variability or measurements of muscle sympathetic nerve activity would have allowed a more comprehensive assessment of autonomic function.

The present study provides further evidence for the occurrence of pure orthostatic cognitive deficits in POTS, particularly in relation to working memory function, and found that these were associated with patient-reported symptom severity. In addition, the results show that patients with neuropathic POTS had elevated norepinephrine concentrations independent of body position, with an excessive increase in the upright body position that was related to heart rate and subjective symptom perception. Acute water intake in patients with neuropathic POTS decreased norepinephrine concentrations and heart rate, and improved symptoms, which in turn was associated with better cognitive performance. In conclusion, the results of the present study indicate that orthostatic cognitive dysfunction in neuropathic POTS is associated with the symptom severity experienced by patients and support the use of bolus water drinking for down-regulation of the sympathetic nervous system as the basis of symptomatic treatment.

## Data availability statement

The raw data supporting the conclusions of this article will be made available by the authors, without undue reservation.

## Ethics statement

The studies involving human participants were reviewed and approved by Kantonale Ethikkommission Bern, Bern, Switzerland. The patients/participants provided their written informed consent to participate in this study.

## Author contributions

BR, RM, and WZ’G designed the research. BR, AH, and WZ’G performed all study procedures. EG and PE performed all laboratory analyses and interpretations. BR and WZ’G did the data analysis. All authors contributed with data interpretation. BR and WZ’G wrote the manuscript. All authors discussed the results and revised the manuscript.
