# Erratum: Brain fog in neuropathic postural tachycardia syndrome may be associated with autonomic hyperarousal and improves after water drinking

**DOI:** 10.3389/fnins.2023.1178850

**Published:** 2023-03-14

**Authors:** Frontiers Production Office

**Affiliations:** Frontiers Media SA, Lausanne, Switzerland

**Keywords:** neuropsychology, cognition, stress, autonomic neuropathy, autonomic dysfunction, dysautonomia

An omission to the funding section of the original article was made in error. The following sentence has been added: “Open access funding was provided by the University of Bern.”

The original article has been updated.

