# Supplementary material for: Brain fog in neuropathic postural tachycardia syndrome may be associated with autonomic hyperarousal and improves after water drinking
Source: Front Neurosci. 2022 Aug 5;16:968725. doi: 10.3389/fnins.2022.968725 (PMC9388780; doi:10.3389/fnins.2022.968725)
Supplement: Supplementary file 1 [file Data_Sheet_1.PDF]

Supplementary Material

1 Material and Methods

1.1 Assessment of plasma catecholamine concentrations

1.1.1 Principle

This method was developed for the multiplex measurement of epinephrine (E) and norepinephrine (NE). The method was adapted from (1), with two main modifications: (a) the preparation of calibrators and of the controls and (b) the replacement of the online solid phase extraction (SPE) by an off-line SPE. These two modifications are presented below.

1.1.2 Calibrators and controls

The *Clincal* commercial calibrator from Recipe (Munich, Germany) was reconstituted in HCl 0.1 M to provide the calibrator A. It was then diluted 1:1 in HCL 0.1 M to provide calibrators B to G. An additional zero calibrator was made of HCl 0.1 M. Calibrators F and G were only used for NE.

Four QC samples were prepared in li-heparin plasma: QC low 1, low 2, medium, and high. QC low 1 was obtained by pooling plasmas with very low NE concentrations (0.25 - 0.38 nM). QC low 2 was obtained by pooling plasmas containing usual catecholamines concentrations (E: 0.05 - 0.33; NE: 1.04 - 4.83). QC medium and high were obtained by spiking plasma with catecholamines in order to obtain concentrations of approximately 40% and 80% of calibrator A, respectively.

1.1.3 Derivatization and off-line SPE

The sample preparation was performed on an Extrahera automation system (Biotage, Uppsala, Sweden). The samples (50 µL of specimen, quality control (QC) sample, or calibrator) were mixed with 50 µL of IS working solution, 850 µL of a solution containing K_2HPO_4 0.5 M and K_2ETDA 4 mM, and 50 µL of a 1:3 mixture of propionic anhydride and acetonitrile (ACN). After 15 min, 900 µL of the reaction mixtures were loaded on an Oasis HLB 5 mg 96-well plate previously conditioned and equilibrated with 500 µL ACN and water, respectively. The wells were washed with 500 µL water and the derivatized catecholamines were eluted with 200 µL of a solution containing 90% of ACN and 0.1% of formic acid.

1.2 UHPLC-MS/MS method validation

The lower limit of quantification (LLOQ) was determined by analyzing five replicate samples containing the IS at levels below the lowest calibrator. For each series of the five replicates, the CV of the measured concentrations and average bias were calculated. The LLOQ was determined as the lowest concentration where the peak of the analyte is identifiable, discrete and reproducible with a CV <20% and average bias <20% with at least five replicates.

Inter-assay imprecision (also known as intermediate precision) and bias were determined by analyzing quintuplicates of the same samples in three different series. The bias were calculated as the mean of the relative difference of the measured *vs*. target concentration.

2 Results

2.1 UHPLC-MS/MS method validation

The LLOQs were determined at 0.040 nM for E and 0.033 nM for NE.

The intra-assay (n = 5) and inter-assay imprecision (n = 3 × 5) evaluated at three concentrations were all below 10% (Table S1). The mean bias were below 15%, or below 20% for the lowest concentrations.

For further details on method validation, please see (1).

3 References

1. Van Faassen M, Bischoff R, Eijkelenkamp K, de Jong WHA, van der Ley CP, Kema IP. In matrix derivatization combined with LC-MS/MS results in ultra-sensitive quantification of plasma free metanephrines and catecholamines. Anal. Chem. 2020;92:9072.

Table S1. Intra-assay (n = 5) and interassay (n = 3 × 5) imprecision and bias of the UHPLC-MS/MS assay.

Nominal concentration (nM)	Intra-assay imprecision (CV%)	Inter-assay imprecision (CV%)	Intra-assay bias (%)	Inter-assay bias (%)
Epinephrine				
0.0702	10%	10%	-13%	-13%
2.80	6%	6%	-12%	-12%
5.53	2%	3%	-15%	-14%
Norepinephrine				
1.035	4%	4%	-17%	-17%
3.99	2%	4%	-13%	-13%
6.95	2%	5%	-11%	-12%
